# Efficacy of A Novel Smart Polymeric Nanodrug in the Treatment of Experimental Wounds in Rats

**DOI:** 10.3390/polym12051126

**Published:** 2020-05-14

**Authors:** Ekaterina V. Silina, Natalia E. Manturova, Vitaliy I. Vasin, Elena B. Artyushkova, Nikolay V. Khokhlov, Alexander V. Ivanov, Victor A. Stupin

**Affiliations:** 1 Department of Human Pathology, I.M. Sechenov First Moscow State Medical University (Sechenov University), Trubetskaya Str, 8, 119991 Moscow, Russia; 2 Department of Plastic and Reconstructive Surgery, Cosmetology and Cell Technologies, N.I. Pirogov Russian National Research Medical University (RNRMU), Ostrovityanova St., 1, 117997 Moscow, Russia; manturovanatali@yandex.ru; 3 Department of Hospital Surgery №1, N.I. Pirogov Russian National Research Medical University (RNRMU), Ostrovityanova St., 1, 117997 Moscow, Russia; vitalikv2010@mail.ru (V.I.V.); stvictor@bk.ru (V.A.S.); 4 Research Institute of Experimental Medicine, Kursk State Medical University, Karl Marx St, 3, 305041 Kursk, Russia; eartyushkova@mail.ru (E.B.A.); nikolay.khokhlov@gmail.com (N.V.K.); 5 Department of Histology, Embryology, Cytology, Kursk State Medical University, Karl Marx St, 3, 305041 Kursk, Russia; anatomy@mail.ru

**Keywords:** nanomedicine, smart polymeric nanodrug, nanoparticles, cerium dioxide, skin wounds, wound healing, experimental study, rat

## Abstract

High-quality and aesthetic wound healing, as well as effective medical support of this process, continue to be relevant. This study aims to evaluate the medical efficacy of a novel smart polymeric nanodrug (SPN) on the rate and mechanism of wound healing in experimental animals. The study was carried out in male Wistar rats (aged 8–9 months). In these animals, identical square wounds down to the fascia were made in non-sterile conditions on the back on both sides of the vertebra. SPN was used for the treatment of one wound, and the other wound was left without treatment (control group). Biocompatible citrate-stabilized cerium oxide nanoparticles integrated into a polysaccharide hydrogel matrix containing natural and synthetic polysaccharide polymers (pectin, alginate, chitosan, agar-agar, water-soluble cellulose derivatives) were used as the therapeutic agent. Changes in the wound sizes (area, volume) over time and wound temperature were assessed on Days 0, 1, 3, 5, 7, and 14. Histological examination of the wounds was performed on Days 3, 7, and 14. The study showed that the use of SPN accelerated wound healing in comparison with control wounds by inhibiting the inflammatory response, which was measured by a decreased number of white blood cells in SPN-treated wounds. It also accelerated the development of fibroblasts, with an early onset of new collagen synthesis, which eventually led to the formation of more tender postoperative scars. Thus, the study demonstrated that the use of SPN for the treatment of wounds was effective and promising.

## 1. Introduction

Cerium oxide (CeO_2_) nanoparticles have been known for a long time and are widely used in technical solutions for the fuel, semiconductor, and chemical industries [1]. The antioxidant effect of CeO_2_, discovered and proven by many researchers, has allowed the development of drugs for use in medicine and veterinary medicine [2]. A particularly large number of works devoted to the study of the effect of using agents with CeO_2_ have appeared in recent years. According to PubMed, over the past 10 years, the number of works devoted to cerium dioxide has increased 3.4 times from 46 publications in 2009 to 156 publications in 2019. This is explained by the fact that oxidative stress plays a leading role in the development and progression of many diseases and pathological conditions, including cardiovascular and cerebrovascular diseases, diabetes, and chronic wounds [2,3,4,5,6].

Wound healing problems will remain relevant for a long time [7,8,9]. This is due to the increasing number of surgeries in the world, as well as domestic and workplace injuries and combat wounds [10,11,12,13,14]. Furthermore, the increase in life expectancy is accompanied by an increase in the prevalence of chronic trophic ulcers of various origin, the healing of which is a clinical problem [15,16,17,18]. The problem of wound healing is especially important in cases when the patients cannot be continuously monitored by clinicians and in cases when there are special requirements for the surgical scar, e.g., in aesthetic surgery [19]. Thus, this highly relevant problem remains unsolved despite the constant emergence of drugs used for wound treatment.

With the aim of solving this problem, the authors created a medical product based on natural and synthetic polymers containing nanoparticles. We aimed to evaluate the medical efficacy of a novel smart polymeric nanodrug (SPN) on the rate and the mechanism of wound healing in experimental animals, along with the assessment of potential further uses of SPN in clinical practice.

Biocompatible citrate-stabilized cerium dioxide nanoparticles were integrated into a polysaccharide hydrogel matrix containing natural and synthetic polysaccharide structural polymers (pectin, alginate, chitosan, agar-agar, water-soluble cellulose derivatives including carboxymethyl cellulose). The cerium dioxide used in the work was synthesized according to the technology developed at the Kurnakov Institute of General and Inorganic Chemistry of the Russian Academy of Sciences (Moscow, Russia). The synthesis process and characteristics of the CeO_2_ nanoparticles used in this work are described in detail in the literature [20,21,22]. The results of the preliminary analysis of the product, as well as the results of experiments that demonstrate its safety and biocompatibility, have been published earlier [23,24,25]. Nanocrystalline CeO_2_ was chosen for the development of a medical product for wound healing because it has low toxicity, is highly biocompatible, and can provide electron transfer by the transition of the valence from Ce^3+^ to Ce^4+^ and back depending on the changing pH value [26,27,28,29]. This is why the medicine is called "smart" and makes CeO_2_ a promising agent for use in nanomedicine [30,31,32,33,34,35]. In particular, it allows one to stabilize the wound environment to neutralize reactive oxygen species or any other free radicals, providing antioxidant, antibacterial, and anti-inflammatory effects [1,26,35,36,37]. The effect of electron transfer can be repeated many times as the wound discharge penetrates through the polysaccharide hydrogel structure directly to the CeO_2_ nanoparticles, or if the wound flora changes. Not only does the developed polysaccharide hydrogel matrix allow one to ensure convenient application of the product to the skin, but it can also provide a prolonged release of nanoparticles from the hydrogel matrix to the affected area, reducing the inflammation and preventing the development of infections, while at the same time stimulating the proliferative activity of fibroblasts. The authors believe that all these factors can potentially create the conditions for the quickest and most effective wound healing.

## 2. Materials and Methods

To assess the medical efficacy of the smart polymeric nanodrug (SPN), which is based on natural and synthetic materials, an experimental control-comparative study was conducted in 30 old white laboratory male Wistar rats (aged 8 to 9 months) weighing at least 350 g (422.5 ± 44.1 g). Wounds of a standardized size were made on the back on both sides of the vertebra of the animals following anesthesia (chloral hydrate, 300 mg/kg) under non-sterile conditions (Patent RF №79701/10.01.2009 [38]). Square wounds with a length of 11 mm, as deep as the fascia, were made. A total of 60 wounds were made. SPN was used to treat one wound, and the other wound was left without treatment (control group). SPN was applied topically once a day on Days 0, 1, 2, 3 4, 5, and 7 of the study by applying it over the wound with a needle-free syringe and filling its volume using 1 mL per cm^2^. The presence of the drug in the wound was monitored daily. In order for the drug to remain in the wounds for the first 5 days, the wounds were covered with a sterile patch. We emphasize that the wounds were inflicted on the backs of animals to avoid them licking the hydrogel. In addition to monitoring the wounds throughout the experiment, animals were kept in separate cages, so additional effects on the wound from other animals were excluded.

The baseline area of the test wounds on the day of surgery, due to size standardization, was statistically the same in the study groups and averaged (median values) 131.2 mm^2^ in the control group and 135.6 mm^2^ in the SPN group (p > 0.05). 

According to the study design, six trigger points were identified: wound modeling—Day 0; changes in the sizes and conditions of the wounds over time—Days 1, 3, 5, 7, and 14. Skin tissue fragments were collected for histological examination on Days 3, 7, and 14 following euthanasia of the rats (by full exsanguination using a puncture of the right ventricle of an anesthetized animal). At each time point, seven wounds of each group were examined with the inspection of three locations (the center and two opposite edges) in each wound. The rest of the animals (n = 7; two rats died on Day 1 of the study, presumably due to anesthesia) were not euthanized until complete regeneration and the development of skin appendages (including fur) on the scar. This allowed us to determine the endpoint of wound healing with the assessment of the scar from the aesthetic viewpoint.

The study was conducted in accordance with the ethical principles of handling laboratory animals “European Convention for the Protection of Vertebral Animals Used for Experimental and Other Scientific Purposes. CETS No. 123”. The study was approved at a meeting of the Regional Ethics Committee of the Federal State Budget Educational Institution of Higher Education at the Kursk State Medical University of the Ministry of Health of Russia (Protocol №5 from November 2, 2017).

Throughout the entire study period, the rats were kept in the standard conditions of the vivarium. Prior to the experiment, the animals were allowed to adapt during a two-week acclimation period (quarantine). The animals were kept in individual cages with a 12:12 h light/dark cycle and had access to food and water ad libitum. The rats received a balanced diet with briquette feeds for laboratory animals (10 g of feed per 100 g of a rat’s body weight, but not more than 50 g of feed per animal per day). The feeds were distributed once daily at the same time simultaneously to all animals. Drinking valves were replaced at the same time with feed distribution.

The study was conducted using current methods of assessment of the drug action (visual inspection, investigations and laboratory tests) according to the “Guideline for Experimental (preclinical) Study of New Pharmacological Substances” developed by the Federal State Institution “Research Center for Expert Evaluation of Medical Products”, Federal Service for Surveillance in Healthcare and Social Development.

Visual inspection of the animals was conducted daily. The examination included the assessment of the general condition of the animals, their motor activity, the response to standard stimuli, and the condition of the skin and the wounds. Evaluation of integral parameters and recording of the results were carried out after weighing the rats. Animals were weighed before conducting any manipulations. Trying to avoid causing any anxiety or pain, a rat was carefully removed from its cage to a metal scale (Supra BSS-4095) and weighed with a precision of 1 g.

**Figure 1:**
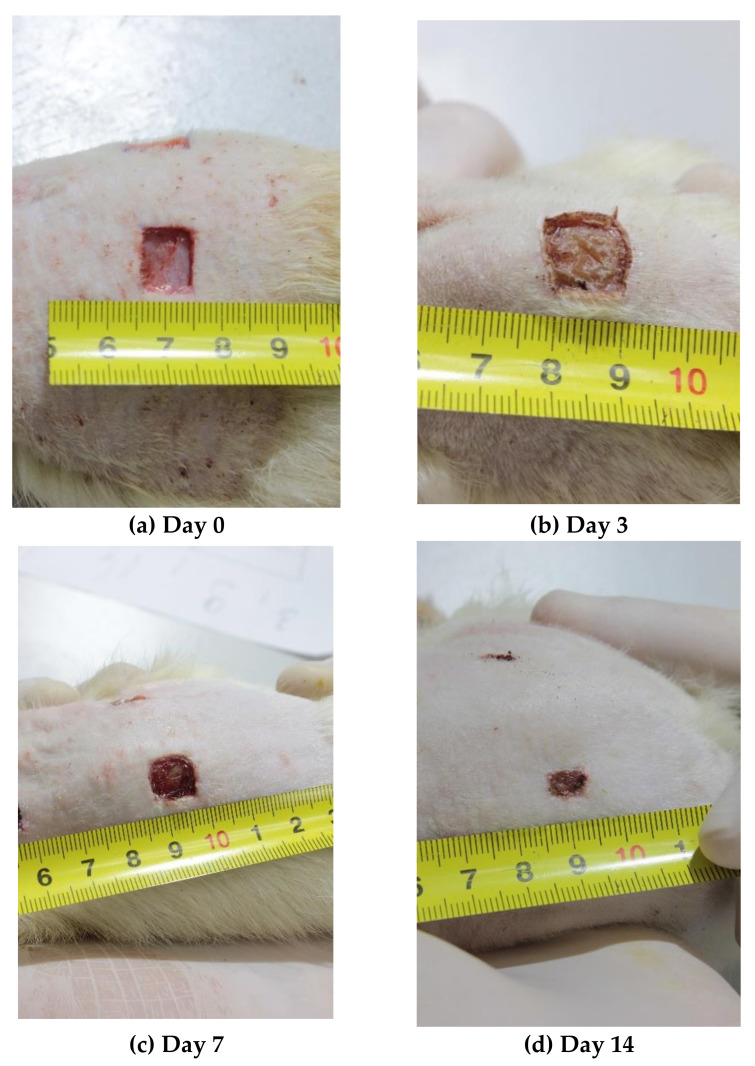
Photographs of the wounds over time (Days 0, 3, 7, 14).

On Days 0, 1, 3, 5, 7, and 14 of the experiment, the photographs of the wounds were taken using a Manfrotto 055 tripod (Manfrotto, Via Valsugana, Cassola, Italy), a Manfrotto MHXPRO-BHQ6 tripod head (Manfrotto, Via Valsugana, Cassola, Italy), and a Canon EOS550D digital camera (Canon, Tokyo, Japan) with a Canon EF-S18-55 lens (Canon, Tokyo, Japan) mounted on the tripod (focal length of 50 mm, distance to the object = 30 ± 3 cm, aperture f = 11, Jpeg digital image format). Lighting of the object was provided using a led searchlight Эpa-50-6500K-M (Era, Moscow, Russia) (power = 50 W, luminous flux = 3500 Lm, color temperature = 6500 K, distance to the object = 1 m). During the shooting, the animal was positioned so that the photographed wound was in the focal plane in the center of the frame. A ruler with millimeter divisions was positioned next to the wound in the field frame using a rotary holder (Figure 1).

To determine the exact planimetry, the area of the wound restricted by its edges was evaluated by analyzing each photograph using JMicroVision 1.2.7 software (free download; Nicolas Roduit University of Geneva, Geneva, Switzerland), and the result was expressed in mm^2^. 

On Day 14, the wound areas were classified based on the healing results as good (≤10 mm^2^), satisfactory (11–30 mm^2^), or unsatisfactory (>30 mm^2^).

The volume of the wound was determined using a standardized drop measurement device (100 drops of distilled water corresponds to 2.554 mL). As estimation of the wound volume could only be performed in immobilized animals in the position where the wound was in the horizontal position for about 1 minute, the measurements were performed in anesthetized animals on Days 0, 3, 7, and 14.

Thermometry of the wound centers was performed using a visual infrared thermometer (Fluke VT02 (FLUKE corporation, Everett, WA, USA) from a distance of 12 ± 2 cm on Days 0, 1, 3, 5, 7, and 14 of the experiment. The temperature of the wound surfaces was recorded in grades of Celsius.

For histological examination, paraffin blocks of the excised skin wound fragments (that had to involve the wound and its edges with visually intact skin and preserved epidermis derivatives) were cut using a M3П-01 microtome (Technonorm Design Bureau, Moscow, Russia). Skin sections (5 μm) were placed on slides (several skin slides obtained from animals of the same batch/term/wound number were placed on the same slide in ascending order of the animal numbers). Following staining with hematoxylin–eosin or van Gieson stains, a Bio Mount HM medium (BioOptica, Milano, Italy) was applied to the slides, which were then covered with coverslips. 

After that, light microscopy of the slides at various magnifications (×40, ×100, ×400) was performed using a Leica CME microscope (Leica Microsystems Inc., Wetzlar, Germany) and an eyepiece camera. The study was blind so the pathologists did not know the group of the wounds they were examining. When assessing the healing stage, the following parameters were considered: the condition of the wound crater, the development of the marginal epithelial healing roll, the number of epidermis layers, and the degree of the wound crater cover and granulations. The amount of granulation tissue in the center and near the edges of the wound was analyzed. When assessing the inflammation stage, the following parameters were considered: the presence and the degree of edema, the presence of blood stagnation in blood vessels, the severity and the gradient of leukocyte infiltration, and the presence, maturity degree and spatial organization of collagen fibers. When conducting morphometry, special attention was paid to the estimation of the density of resident cells in the granulation tissue layers (total number of fibroblasts and fibrocytes). The rest of the cells were non-resident (total number of granulocytes, macrophages, lymphocytes, and monocytes recruited to the inflammation site).

**Statistical data analysis** was conducted using Statistical Package for the Social Sciences SPSS 23.0 software (IBM Company, New York, NY, USA). The differences were considered statistically significant when p < 0.05. Descriptive statistics of quantitative continuous variables are presented as mean values (M) and standard deviations (±SD) for normally distributed data, and as medians (Me), upper Q_3_ (75%), and lower Q_1_ (25%) quartiles for non-normally distributed data. The Mann–Whitney U test was used to compare two independent non-parametric samples. The Wilcoxon test was used to compare two dependent non-parametric samples. Qualitative parameters were compared using the χ2 test (analysis of contingency tables). 

## 3. Results

### 3.1. Changes in the Sizes of Wounds over Time

In the absence of treatment, the wound area on Day 1 demonstrated a 1.21-fold increase (p = 0.01) up to 158.3 mm^2^, followed by a return to baseline by Day 3 (p = 0.649). Regression of the area of the control wound by an average of 10% to 118.1 mm^2^ (p = 0.039) was observed only by Day 5 (not in all wounds). The area of the control wounds decreased by Day 7 to an average of 99.4 mm^2^ (p = 0.003), and to 22.1 mm^2^ (p = 0.003) by Day 14. Thus, on the day of modeling, the area of the control wound was 5.9 times higher than that on Day 14 of the study.

The use of SPN contributed to more rapid and better wound epithelization, which manifested in a gradual decrease in the wound area on average from 135.6 mm^2^ on Day 0 to 9.1 mm^2^ on Day 14 (14.9-fold difference, p < 0.001) as well as the absence of the period of the wound area extension. By Day 1, the area of the SPN group wounds remained unchanged relative to Day 0, with the mean value of 134.5 mm^2^. By Day 3, the area of the wounds significantly decreased to 123.4 mm^2^ (p < 0.01; the initial size being 1.1 times the size on Day 3). By Day 5, the mean wound area in SPN groups was 108.7 mm^2^, i.e., on the day of modeling, the wound area was 1.25 times the initial area (p < 0.01). By Day 7, the mean wound area was 85.2 mm^2^, i.e., on Day 0, it was 1.59 times the initial area (p < 0.01). On Day 14, the area of the SPN group wounds varied within the range of 1.77–26.30 mm^2^, and the control group had the higher range of 5.17–63.85 mm^2^. 

There was a statistically significant difference between the groups in terms of the wound area as early as on Day 1, as well as on Days 5, 7, and 14 of the study. On Day 1, the area of the control group wounds was 1.17 times that in the SPN group (p < 0.01), on Day 5 it was 1.10 times (p < 0.05), on Day 7 it was 1.17 times (p < 0.05), and on Day 14 it was 2.43 times (p < 0.01) that in the SPN group. Thus, the study demonstrated the advantages of SPN in respect to the changes in the wound areas over time compared to the wounds left without treatment under scabs (Table 1).

The result of healing was good (the wound area ≤10 mm^2^) by the end of Week 2 in 14.3% of cases in the control group and 57.1% in the SPN group (4-times more often), and a satisfactory outcome was recorded in 57.1% and 42.9% of cases in the control and SPN groups, respectively. An unsatisfactory outcome (a wound area of >30 mm^2^) by Day 14 was recorded only in the control group (28.6%). Analysis of contingency tables demonstrated the efficacy of SPN (χ2 Pearson = 7.89, p = 0.020). However, full epithelization of the wounds was not observed in any case.

Analysis of changes in the wound area (expressed in percent) showed that the median wound area in the control group significantly expanded (by 17.7%) in the control group by the end of Day 1. In a quarter of cases, the area of untreated wounds increased by more than a third compared to baseline (p < 0.01). The use of SPN contributed to the inhibition of the inflammatory response; the mean reduction of the wound area compared to the day of surgery was 1.2% (p > 0.05). There was a 19% difference between the groups on Day 1 (p < 0.01). The difference between the groups was observed at all time points. Thus, on Day 3, the control wound area remained unchanged compared to Day 0; the mean reduction being only 0.5%. In the SPN group, the wound area decreased by 7.9% on Day 3; more than 75% of the wounds in this group were smaller compared to the day of modeling. On Day 5, the area of control wounds decreased by 9.4% compared to Day 0; in the SPN group, the area of wounds decreased by 25.6% (which was 2.72 times than that in the control group; p < 0.01). On Day 7, the area of the control wounds decreased by 23.9%; in the SPN group, the area of wounds decreased by 38.8% (which was 1.62 times more than that in the control group; p < 0.01). On Day 7, almost 20% of the wounds left without treatment demonstrated negative changes (expansion) compared to Day 0, while 100% of wounds in the SPN group showed a reduction. On Day 14, the area of control wounds decreased by 82.9%; in the SPN group, the area of wounds decreased by 94.7% (p < 0.01) (Table 2). 

The volume of wounds in mL on the day of modeling was comparable in the study groups with the mean values of 0.405 mL in the control group and 0.422 mL in the SPN group (p > 0.05). On Day 3, tissue regeneration in the wound bottom reduced the size of the wound to 0.204 mL in the control group (the difference being 1.99-fold). The use of SPN significantly reduced (2.99-fold) the wound volume by Day 3 (to 0.141 mL on average); and the volume of control wounds on Day 3 was 1.44 times that in the SPN group (p = 0.040). On Day 7, the mean volume of the control wounds was 0.115 mL. This was 9.58 times the volume of the wound in the SPN group (0.012 mL; p = 0.001). Thus, the use of SPN resulted in the acceleration of reduction of the sizes and depth of the wounds with the volume reduction of 30.9% by Day 3 and 89.6% by Day 7 (Table 3). On Day 14, the volume of wounds was not estimated due to the beginning of epithelization and, as a result, the superficial nature of the wounds.

The rates of wound healing were comparable in the absence of treatment in both groups. By Day 21, full epithelization was observed in 28.6% and 42.9% of the wounds in the control group and the SPN group, respectively; by Day 28, it was observed in 57.1% of cases in both groups; all wounds healed by Day 35. In the SPN group, more aesthetically-pleasing scars were observed.

### 3.2. Changes in the Wound Temperature over Time

**Figure 2:**
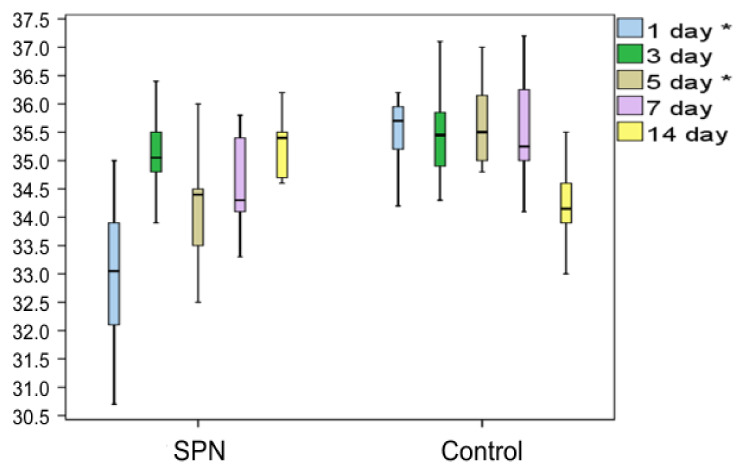
Changes in the temperature of the wound center (°C). * indicates a difference between the groups on Day 1 and Day 5 p < 0.05 (the Mann–Whitney test).

Analysis of the changes in the wound center temperature demonstrated an anti-inflammatory effect of SPN, which manifested in lower temperature ranges of the wound center on Day 1 and Day 5 compared to the control wounds (Figure 2).

### 3.3. Histological Examination Findings

Wound regeneration in the SPN group was better with respect to the rate of formation of the marginal epithelial zone, restoration of the number of layers and organization of the epidermal layer, the emergence and distribution of points of growth of the epidermal derivatives, the rate of changes in the phases of healing (from exudative to proliferative) taking into account qualitative and quantitative characteristics of the inflammatory infiltrate at different time points, the distribution of the infiltrate in the granulation tissue layers and outside them, the development and severity of the signs of remodeling in the dermis fibers, and the rate of maturation of collagen fibers in the granulation tissue. Wound regeneration was worse in the control wounds in respect of all above-mentioned parameters.

**Figure 3:**
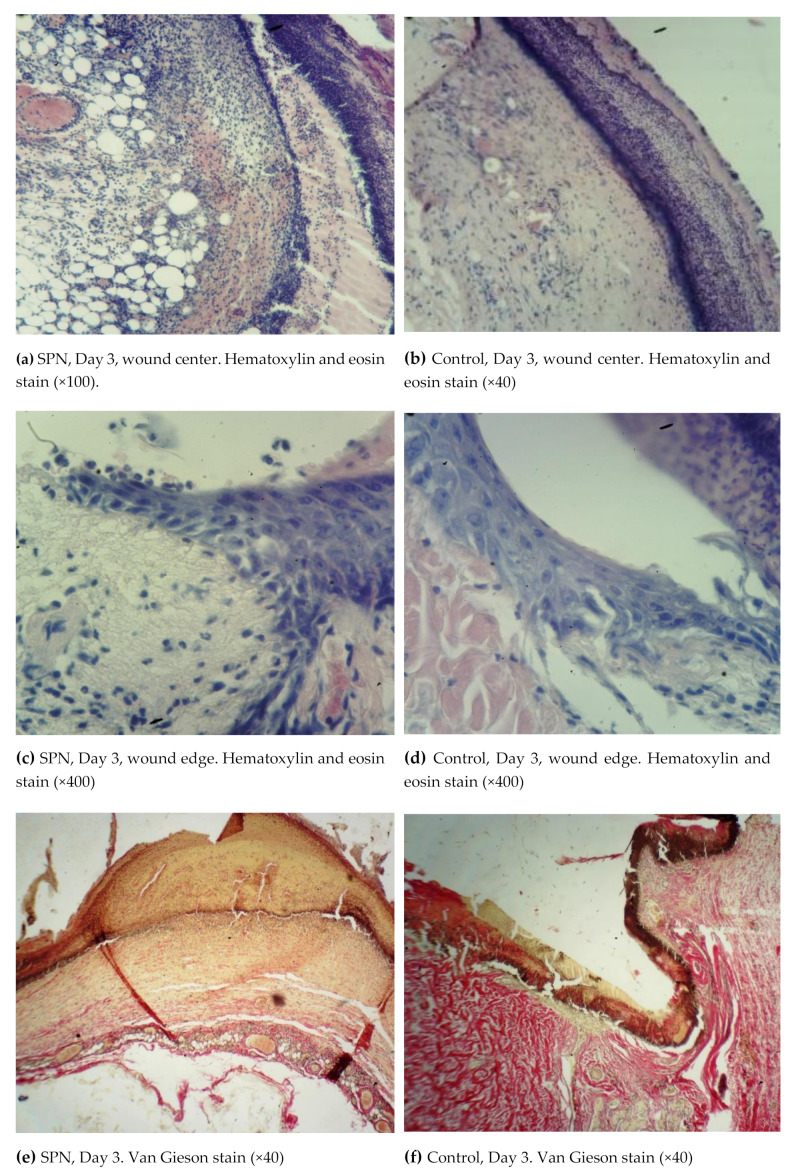
Morphological patterns of the SPN group wounds (**a**,**c**,**e**) and the control group wounds (**b**,**d**,**f**) on Day 3 of the study.

Thus, on Day 3, all animals in both groups had an exudative phase of inflammation in both the bottom and the edges of the wounds. All wounds demonstrated a significant tissue deficiency (the wound was not completely filled with granulations). The rate of regeneration of the SPN group wounds was higher than in the control group with respect to the granulation tissue thickness. The scab and the residual amount of the ointment were infiltrated with neutrophils. Immature collagen was found in 85.7% of the SPN group wounds on Day 3; the amount of this collagen was poorly pronounced and moderately pronounced in 28.6% and 57.1% of cases, respectively. In the control group, immature collagen was poorly expressed in 57.1% of cases, and no collagen was detected in 42.9% of the wounds. There was swelling in the connective tissue of all wounds; however, the most pronounced edema was observed in the control wounds. Venous hyperemia was observed in all wounds. It was highly pronounced in the control group wounds, where there was maximum swelling (Figure 3).

On Day 3, cell parameters recorded in the layer of vascular loops were studied (other layers had not yet developed by that time). By Day 3, white blood cells were predominant in the bottom and especially in the edges of the wounds (the inflammation phase), and leukocytic infiltration was observed in 100% of the wounds in both groups. However, the percentage of white blood cells per unit area was higher in the control group (on average 63.0% vs. 59.1% per mm^2^), and the nuclei of resident cells (fibroblasts) in the wound bottom were 1.11 times larger in the SPN group than in the control group (41% vs. 37% per mm^2^ of the wound bottom area). The mean total number of fibroblasts on Day 3 at the bottom of the control group wounds was 118 [109:121] per 1 mm^2^, and in the SPN group, it was 131 [126:139] per 1 mm^2^ of the tissue section (p < 0.05). This explains the more rapid epithelization of the SPN group wounds. As early as Day 3, epithelization with the formation of the marginal epithelial roll was observed in 100% of the SPN group wounds. Marginal epithelization was observed in 71.4% of the control group wounds, while 28.6% of the wounds showed no signs of epithelization. In these cases, the area of the wounds was larger on Day 3 compared to Day 0.

**Figure 4:**
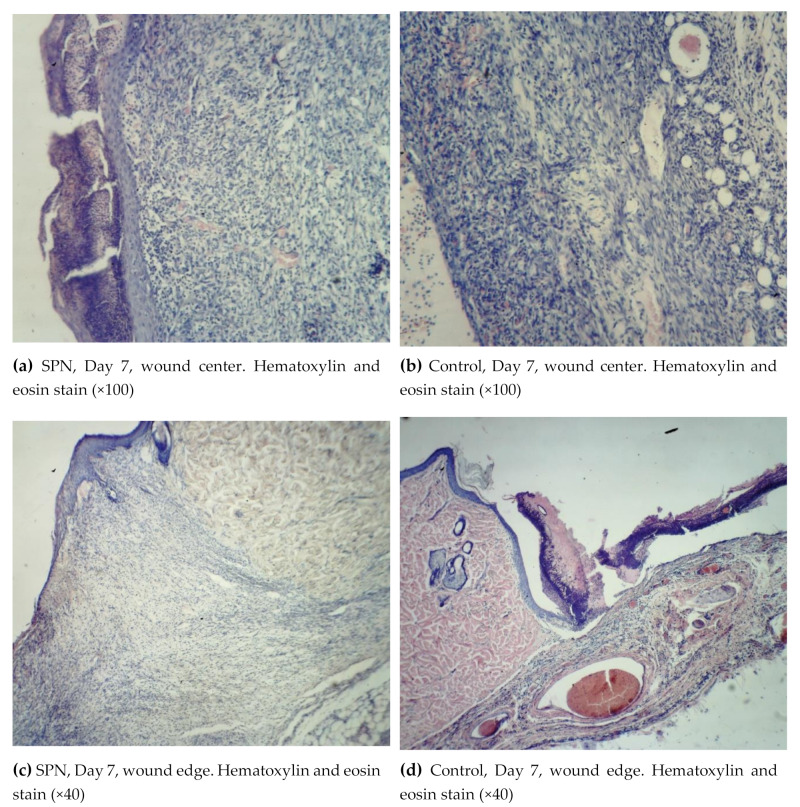

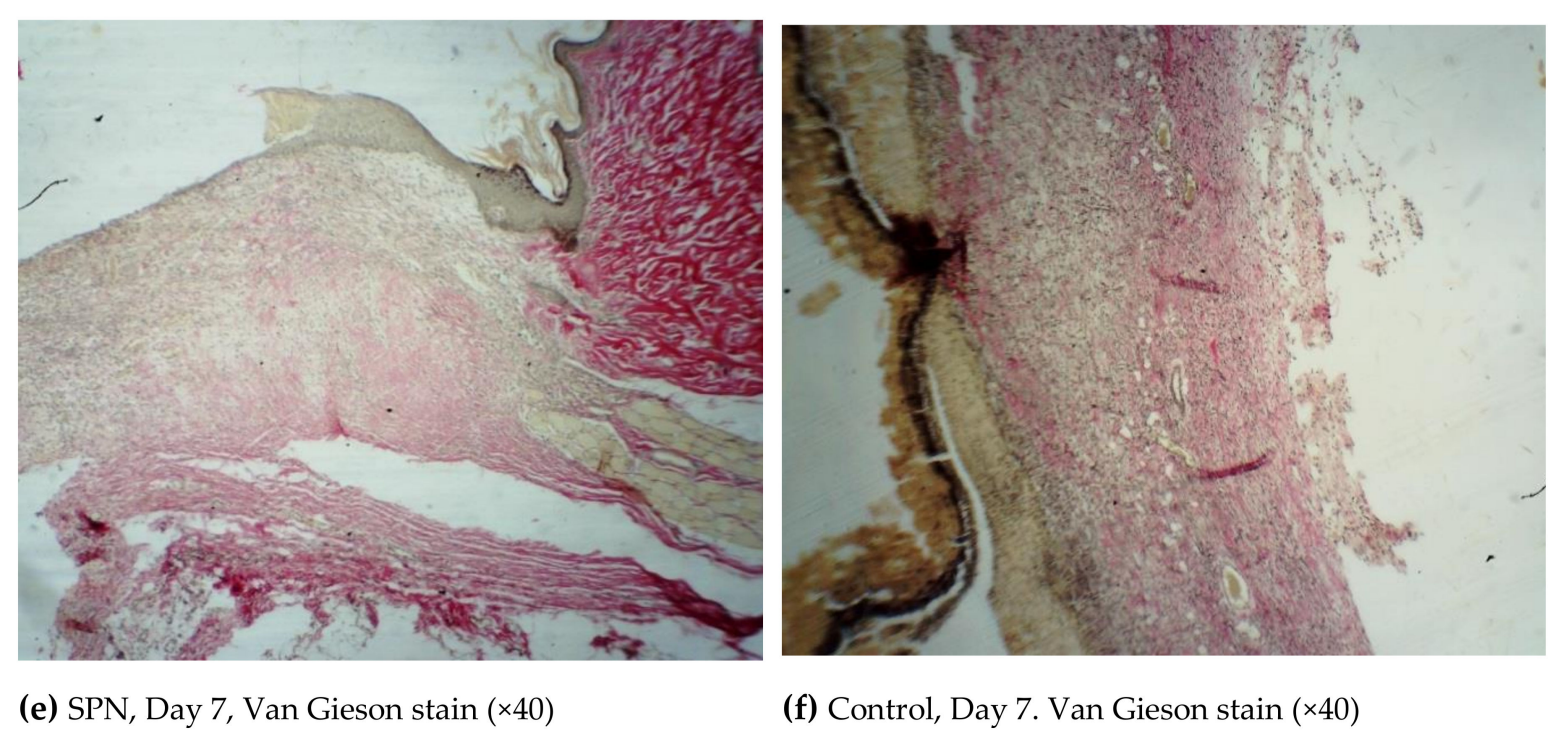
Morphological patterns of the SPN group wounds (**a**,**c**,**e**) and the control group wounds (**b**,**d**,**f**) on Day 7 of the study.

On Day 7, all wounds demonstrated marginal epithelization, pronounced angiogenesis, collagen fibers of various degrees of maturity, granulation tissue layers (>2), and 2–3 epidermis layers (Figure 4). 

On Day 7, the epidermis was present in all wounds; however, in 42.9% of the SPN group wounds, there were signs of epidermis splitting and gel incorporation, which was not observed in the control group wounds. In most cases, the epidermis on the edges of the wounds consisted of three layers, then towards the center of the wounds there were two layers and then the epidermis gradually thinned towards the scab. The more pronounced structure of the epidermis was observed in the SPN wounds compared to the control group. In all SPN group wounds, there were three distinctive layers of the epidermis. In the control group, there were three layers of the epidermis in 42.9% of wounds; the rest of the wounds (57.1%) had only two layers of the epidermis (p < 0.05). The control wounds demonstrated the phenomenon of a tissue defect, which is when the wound looks like a crater with its edges covered with a newly-formed epithelium. In the SPN group wounds, the tissue defects were better filled with granulations. Swelling, dilation of lymphatic capillaries, and formation of cystic cavities of various sizes were observed in the edges of all control group wounds and in some wounds in the SPN group. Swelling was observed in all wounds; however, it was less pronounced in the SPN group. The granulation fibers in all cases were thin parallel collagen fibers with fibroblasts between them.

An important feature of the SPN use was the better development of fibroblasts that synthesize the extracellular matrix, collagen precursors, and elastin proteins on Day 7. Cell granulations were present in all skin layers in half of the SPN group wounds, while in the control group wounds, no horizontal layer of fibroblasts was observed on Day 7. The rest of the layers (vascular loops, vertical vessels, maturing layer) were observed in all wounds in both groups. The vascular loops layer was less pronounced, which predominantly manifested in the areas where the scab and the SPN gel lasted a long time. The mean total number of fibroblasts and fibrocytes on Day 7 at the bottom of the control group wounds was 442 [345:446] per 1 mm^2^, and in the SPN group, it was 549 [359:701] per 1 mm^2^ (p < 0.05). The mean ratios of resident to non-resident cells in the control group and the SPN group were 1:1.2 and 1:1.4, respectively.

**Figure 5:**
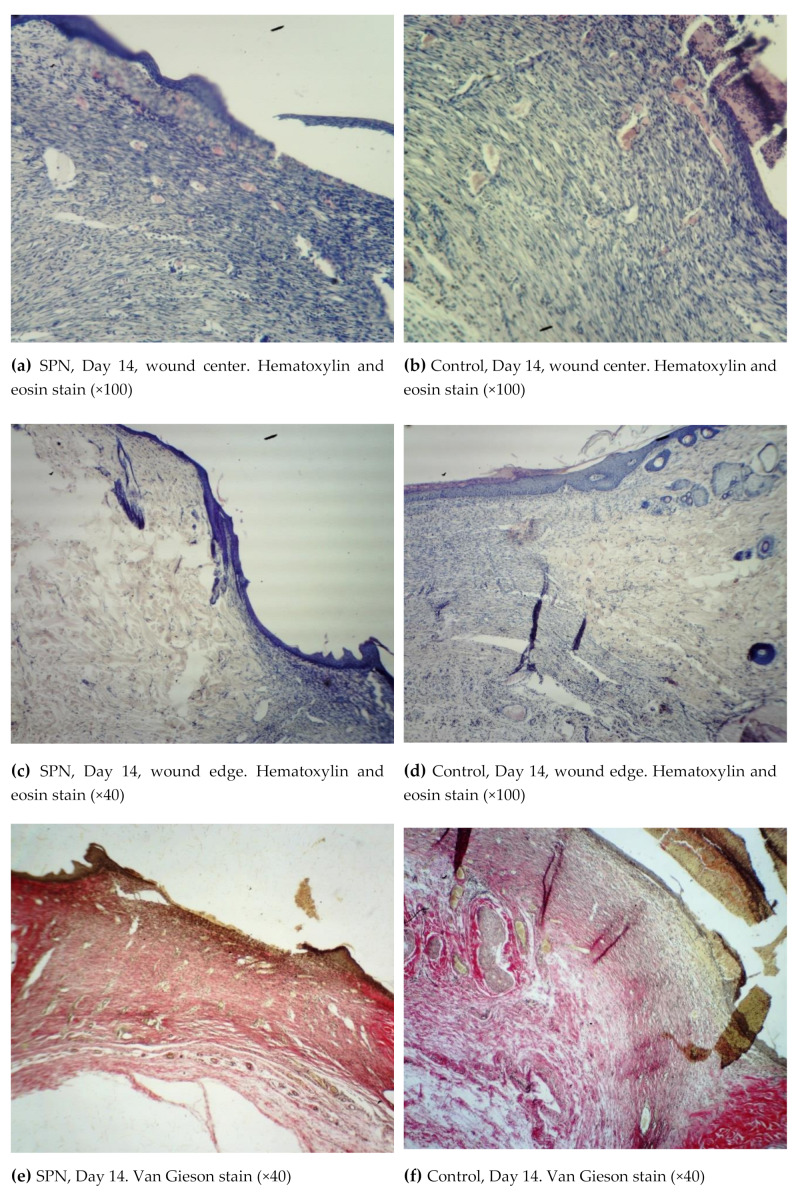
Morphological patterns of the SPN group wounds (**a**,**c**,**e**) and the control group wounds (**b**,**d**,**f**) on Day 14 of the study.

By Day 14, all three layers of the epidermis were present in all groups. In all groups, epithelization, an increased amount of collagen, and signs of neoangiogenesis of various degrees were observed; however, the morphological signs of wound healing were more pronounced in the SPN group (Figure 5).

By Day 14, a multilayer epithelium developed on the top of the granulation tissue in all wounds. It was the most mature in the SPN group wounds, which demonstrated lymphocytic infiltration in all layers of the granulation tissue (the most pronounced in the subepidermal layer). There was a layer of horizontal fibroblasts (100% of wounds in both groups), the maturating layer (100% of wounds in both groups), and a layer of vertical vessels (85.7% and 28.6% in the SPN and control groups, respectively). This explained the difference in neoangiogenesis. On Day 14, neoangiogenesis was more pronounced in 85.7% and 28.6% of the SPN and control group wounds, respectively (p < 0.05). The mean total numbers of resident cells in all layers on Day 14 in the bottom of the wounds were 343 [307:369] per 1 mm^2^ and 548 [480:603] per 1 mm^2^ in the control and SPN groups, respectively (p < 0.05).

On Day 14, fibrous stroma in all wounds in both groups consisted of isolated fibers or bundles of thin, mainly immature, collagen fibers. In the SPN group, 100% of wounds demonstrated a predominance of immature collagen with the presence of mature collagen only in 57.1% of cases. In the rest of the cases (42.9%), the SPN group wounds showed no mature collagen. In the control group wounds, the degree of collagen maturity was higher; all control group wounds demonstrated mature collagen; equal amounts of immature and mature collage were observed in 71.4% of cases, and the predominance of immature collagen was recorded in 28.6% of the wounds (p < 0.05). This explained the development of imperfect scars in the control group and more delicate scars in the SPN group.

## 4. Discussion

Our study showed that the use of a novel smart polymeric nanodrug (SPN) accelerated wound healing compared to the control group, even in old animals. On Day 1, the area of the control group’s wounds was 1.17 times that in the SPN group; on Day 7, it was 1.17 times; and on Day 14, it was 2.43 times that in the SPN group. Acceleration of the regeneration rate is due to inhibition of the inflammatory reaction, which is evident by the decreased number of non-resident cells in the study group. Thus, our results are consistent with the data of Reference [39], which previously also showed accelerated wound healing. Regarding the density of the forming scar obtained by this researcher, our data explains this effect by faster maturation of collagen due to the accelerated proliferation of fibroblasts in the wound. The presence of a polymer gel in cerium dioxide nanoparticles with changing valency in the wound ensures the environment required for regeneration as a result of its anti-inflammatory, antioxidant, antimicrobial, and proregenerative effects. The decrease in the active forms of oxygen can be judged by the decrease in the number of non-resident cells (leukocytes), which are the main producers of free radicals in the wound, which is consistent with the conclusions from different groups of scientists [39,40,41]. The positive effects of CeO_2_ that are relevant for regenerative medicine are consistent with the results of other studies [19,20,21,22,23,24,27,42].

We observed active neoangiogenesis in the SPN group wounds, which provided nutrition to the entire pool of regenerating epithelial cells. An increase in neoangiogenesis occurred in the main group of animals without additional administration of L-arginine, which, according to Reference [42], improved microcapillary blood flow. In addition, since L-arginine was not used additionally in our study, it becomes obvious that NO is not the only trigger mechanism for improving the trophism of the wound cell pool.

The inhibition of inflammation allows for a greater amount of the intercellular substance at the early stage of collagen synthesis and, thus, space for a higher number of regenerating cells. At the same time, the rate of collagen maturation slows down and the differentiation of epithelial layers occurs more evenly, allowing for a more delicate postoperative scar, which is especially important for plastic surgery and aesthetic medicine. We cannot reasonably speak about the contribution of chitosan to the wound healing process, as is discussed in Reference [41], since the gel we used was multicomponent, although it also contained chitosan, and we also determined the anti-inflammatory effect when using CeO_2_ nanoparticles. However, we must agree with Reference [42] that work in this direction should continue with a change in both CeO_2_ synthesis methods and the more accurate and complex biological models used.

## 5. Conclusions

The data obtained in the study allow us to conclude that earlier formation of immature collagen with a slowdown in the rate of collagen maturation is a marker of the formation of a good aesthetic postoperative result. On the contrary, the delayed onset of collagen formation with rapid collagen maturation is a predictor of the formation of a rough, atrophic, or keloid scar (a poor aesthetic result).

The results of this study indicate that SPN is a promising agent and that its efficacy should be analyzed in comparative studies with various clinically-used drugs. Further research on the use of SPN (provided that positive results are obtained) will include treatment of purulent wounds, wounds in hypercapnia, trophic arterial ulcers, and wound defects with microcapillary bed damage in patients with diabetes mellitus.

## Figures and Tables

**Table 1:** Changes over time in the area of wounds in the control and SPN groups (mm^2^).

Day	Control	SPN	p (Mann–Whitney)
0	131.2 113.3/147.6	135.6 121.1/148.8	0.358
1	158.3 # 137.9/175.9	134.5 124.1/143.5	0.001 *
3	132.1 115.3/142.6	123.4 # 115.7/134.2	0.213
5	118.1 # 109.0/134.3	108.7 # 97.6/113.5	0.017 *
7	99.4 # 80.0/111.4	85.2 # 74.2/96.9	0.048 *
14	22.1 # 15.5/30.6	9.1 # 5.4/13.7	0.001 *

Note: The data are presented as follows: 1st line, median; 2nd line, Q1/Q3. * difference between the groups, p < 0.05 (Mann–Whitney U test). # difference in the parameter changes in a specific group compared to Day 0, p < 0.05 (Wilcoxon signed-rank test).

**Table 2:** Changes in proportions of the area of wounds compared to Day 0 in both groups (%).

Day	Control	SPN	p (Mann–Whitney)
1	17.7 # 11.8/33.3	−1.2 −13.4/9.1	<0.001 *
3	−0.5 −7.5/11.1	−7.9 # −14.7/−1.0	0.008 *
5	−9.4 # −13.9/−0.5	−25.6 # −29.8/−13.3	0.002 *
7	−23.9 # −39.3/−0.4	−38.8 # −46.5/−31.3	0.021 *
14	−82.9 # −86.3/−73.8	−94.7 # −96.1/−89.5	<0.001 *

Note: The data are presented as follows: 1st line, median; 2nd line, Q1/Q3. * difference between the groups, p < 0.05 (Mann–Whitney U test). # difference in the parameter changes in a specific group compared to Day 0, p < 0.05 (Wilcoxon signed-rank test).

**Table 3:** Changes in the volume of wounds in both groups (mL).

Day	Control	SPN	p
0	0.405 0.360/0.514	0.422 0.40/0.511	0.305
3	0.204 0.179/0.255	0.141 0.115/0.189	0.040 *
7	0.115 0.102/0.128	0.012 0/0.051	0.001 *

Note: The data are presented as follows: 1st line, median; 2nd line, Q1/Q3. * difference between the groups, p < 0.05 (Mann–Whitney U test).
